# Characteristics of inflammatory factors and lymphocyte subsets in patients with severe COVID‐19

**DOI:** 10.1002/jmv.26070

**Published:** 2020-06-09

**Authors:** Ming Ni, Fang‐Bing Tian, Dan‐Dan Xiang, Bing Yu

**Affiliations:** ^1^ Department of Infectious Diseases, Tongji Hospital, Tongji Medical College Huazhong University of Science and Technology Wuhan China; ^2^ Department of Pathogen Biology, School of Basic Medicine, Tongji Medical College Huazhong University of Science and Technology Wuhan China

**Keywords:** COVID‐19, lymphopenia, proinflammatory, severe pneumonia

## Abstract

To investigate the inflammatory factors and lymphocyte subsets which play an important role in the course of severe coronavirus disease 2019 (COVID‐19). A total of 27 patients with severe COVID‐19 who were admitted to Tongji Hospital in Wuhan from 1 to 21 February 2020 were recruited to the study. The characteristics of interleukin‐1β (IL‐1β), IL‐2 receptor (IL‐2R), IL‐6, IL‐8, IL‐10, tumor necrosis factor‐α (TNF)‐α, C‐reactive protein (CRP), serum ferritin and procalcitonin (PCT), and lymphocyte subsets of these patients were retrospectively compared before and after treatment. Before treatment, there was no significant difference in most inflammatory factors (IL‐1β, IL‐2R, IL‐6, IL‐8, IL‐10, CRP, and serum ferritin) between male and female patients. Levels of IL‐2R, IL‐6, TNF‐α, and CRP decreased significantly after treatment, followed by IL‐8, IL‐10, and PCT. Serum ferritin was increased in all patients before treatment but did not decrease significantly after treatment. IL‐1β was normal in most patients before treatment. Lymphopenia was common among these patients with severe COVID‐19. Analysis of lymphocyte subsets showed that CD4+ and particularly CD8+ T lymphocytes increased significantly after treatment. However, B lymphocytes and natural killer cells showed no significant changes after treatment. A pro‐inflammatory response and decreased level of T lymphocytes were associated with severe COVID‐19.

## Highlights

- A pro‐inflammatory response, particularly the level of IL‐2R, IL‐6, TNF‐α and CRP, were associated with severe COVID‐19. The SARS‐CoV‐2 infection affect primarily T lymphocyte, particularly CD8+ T cells.

## 1 INTRODUCTION

Coronavirus disease 2019 (COVID‐19), which first appeared in Wuhan, China, in December 2019, has rapidly spread all over the world.^1^, ^2^ The World Health Organization characterized COVID‐19 as a pandemic on 11 March 2020. At the time of writing, patients with COVID‐19 had been identified in more than 200 countries worldwide. Up to 22 May 2020, the number of confirmed cases in China had reached 84 522, of which 4645 individuals had died and 79 738 had been cured. In addition to China, the number of confirmed cases in other countries had reached 5 146 723, with 330 959 individuals dying.

COVID‐19 is caused by severe acute respiratory syndrome coronavirus 2 (SARS‐CoV‐2), and is the third highly pathogenic coronavirus to arise, following the SARS‐CoV and the Middle East respiratory syndrome‐CoV. In general, COVID‐19 is an acute resolved disease but it can also be deadly, with a 2.3% case fatality rate.^3^ Currently, the pathogenesis of COVID‐19 is still poorly understood. Lung biopsy samples have shown evidence of acute respiratory distress syndrome (ARDS), which greatly resembles that seen in patients with SARS and MERS.^5^, ^6^ One of the main mechanisms for ARDS in SARS‐CoV and MERS‐CoV infection is the cytokine storm: a deadly uncontrolled systemic inflammatory response resulting from the release of large amounts of proinflammatory cytokines and chemokines by immune effector cells.^7^, ^8^, ^9^, ^10^ A cytokine storm triggers a violent attack by the immune system on the body, causing ARDS and multiple organ failure, and finally, leading to death. However, the main cytokines and chemokines involved in SARS‐CoV and MERS‐CoV infections differ.^7^, ^9^ In COVID‐19, it has been reported that patients being treated on intensive care units (ICU's) have higher plasma levels of interleukin‐2 (IL‐2), IL‐7, IL‐10, granulocyte‐colony stimulating factor, interferon γ‐inducible protein‐10, monocyte chemoattractant protein‐1, macrophage inflammatory protein‐1A, and tumor necrosis factor‐α (TNF‐α) compared to non‐ICU patients.^2^ Regarding other inflammatory factors, elevated IL‐6, serum ferritin, and C­‐reactive protein (CRP) have been most commonly reported in patients with COVID‐19.^11^

A cytokine storm is also associated with apoptosis of lymphocytes, leading to severe and transient lymphopenia.^12^, ^13^, ^14^, ^15^ Lymphopenia is a common feature in patients with COVID‐19 and might be a critical factor associated with disease severity and mortality.^2^, ^11^, ^16^ One of the most recent reports has shown that the number of CD4^+^ and CD8^+^ T cells in the peripheral blood of a SARS‐CoV‐2‐infected patient is significantly reduced, whereas the status of CD4^+^ and CD8^+^ T cells are excessive activation.^4^

With increasing evidence on the key pathophysiological role of inflammatory factors in patients with COVID‐19, immunomodulatory agents including corticosteroids, tocilizumab, and lucitanib have been considered for use in clinics. However, more laboratory and clinical evidence for their use are needed.

In this study, the characteristics of several inflammatory factors (IL‐1β, IL‐2 receptor (IL‐2R), IL‐6, IL‐8, IL‐10, TNF‐α, CRP, serum ferritin, and procalcitonin [PCT]) and lymphocyte subsets of 27 patients with severe COVID‐19 patients were examined. We aimed to find appropriate targets for early intervention in patients with severe COVID‐19 by comparing relevant laboratory indicators before and after treatment. We found that levels of IL‐2R, IL‐6, TNF‐α, and CRP decreased significantly after corticosteroid therapy, followed by IL‐8, IL‐10, and PCT. Analysis of lymphocyte subsets showed that CD4^+^ and particularly CD8^+^ T lymphocytes increased significantly after treatment. However, B lymphocytes and natural killer (NK) cells showed no significant change after treatment.

## 2 MATERIALS AND METHODS

### 2.1 Patients and samples

We recruited 27 patients (14 male and 13 female, age 33‐83 years, and median age 60 years) who were confirmed to have COVID‐19 by Real‐time Fluorescent RT‐PCR Kit, and who had been admitted to Tongji Hospital from 1 to 21 February 2020. According to the Diagnosis and Treatment Protocol of New Coronavirus Pneumonia (trial version 7) from the National Health Commission of China,^17^ all of the patients were defined as having severe pneumonia (ie, adolescents or adults with fever or respiratory symptoms with imaging findings of viral pneumonia, plus one of the following signs: respiratory rate > 30 breaths/min, SpO_2_ < 93% on room air, or PaO_2_/FiO_2_ ≤ 300 mm Hg, patients whose pulmonary imaging progresses greater than 50% in 24 to 48 hours were managed as severe pneumonia). Five patients had type 2 diabetes and eight had hypertension. Patients with a history of hematologic disease, autoimmune disease, or tumors were excluded from the study.

All of the patients received supplemental oxygen. All of the patients were given abidol (200 mg, orally [PO] tid) and five patients were given lopinavir/ritonavir (400/100 mg, PO bid) together as antiviral therapy. Moxifloxacin (0.4 g, intravenously [IV], qd) was used to prevent secondary infection. Methylprednisolone (40 mg, IV, qd or q12h) was administered to attenuate lung inflammation and was given for no more than 1 week. Blood laboratory tests and chest computed tomography were performed every 5 to 7 days. All 27 patients experienced improvements in their condition and as of 12 March 2020, eight male patients and nine female patients had been discharged from hospital. Blood samples of the patients were collected before treatment and after the condition improved significantly (ie, body temperature is normal for more than 3 days, respiratory symptoms improve obviously, and pulmonary imaging shows obvious absorption of inflammation).

### 2.2 Ethics approval

This study received ethical approval from the Medical Ethics Committee of Tongji Hospital, Tongji Medical College, Huazhong University of Science and Technology. All participants gave written informed consent, and the study was carried out in accordance with the Declaration of Helsinki.

### 2.3 Cytokine, CRP, serum ferritin, and PCT detection

Levels of IL‐1β, IL‐2R, IL‐8, IL‐10, and TNF‐α were measured using the IMMULITE 1000 Immunoassay system (Siemens Healthcare Diagnostics Products Limited). Levels of IL‐6 and PCT were determined by electrochemiluminescence immunoassay (Cobas E601; Roche, Basel, Switzerland). CRP and serum ferritin were measured using latex‐enhanced immunoturbidimetry (Cobas 8000; Roche). All procedures were carried out according to the manufacturers’ instructions.

### 2.4 Lymphocyte subset count

The total number of lymphocytes in peripheral blood was counted using a hemocytometer. The percentages of CD3^+^CD4^+^CD8^−^ T lymphocytes, CD3^+^CD4^−^CD8^+^ T lymphocytes, CD3^−^CD19^+^ B lymphocytes, and CD3^−^CD16^+^CD56^+^ lymphocytes among the total lymphocytes were obtained as follows: (a) 100 μL of whole blood was diluted with 400 μL of Iscove's modified Dulbecco's medium; (b) the cells were labeled with antibodies (anti‐CD45, anti‐CD3, anti‐CD4, anti‐CD8, anti‐CD16, anti‐CD19, and anti‐CD56); (c) the cells were fixed and permeabilized; and (d) the cells were analyzed using FACSCanto flow cytometer. The absolute numbers of different lymphocyte subsets were calculated by multiplying the percentages by the total lymphocyte count (CD4^+^ T lymphocytes count = total lymphocyte count × CD3^+^CD4^+^CD8^−^%, CD8^+^ T lymphocytes count = total lymphocyte count × CD3^+^CD4^−^CD8^+^%, B lymphocytes count = total lymphocyte count × CD3^−^CD19^+^%, and NK cell count = total lymphocyte count × CD3^−^CD16^+^CD56^+^%).

### 2.5 Statistical analysis

All statistical analyses were performed using SPSS 22.0 (SPSS Inc, Chicago, IL). Data are expressed as mean ± standard deviation and were compared using the independent‐samples *t* test. A *P* < .05 was considered statistically significant.

## 3 RESULTS

### 3.1 Lymphocyte subsets and most inflammatory factors were not significantly different between male and female patients

The median age of male and female patients was 61 and 60 years, respectively. Before treatment, lymphocyte subsets and inflammatory factors were evaluated (Table 1). Lymphopenia was seen in 71.4% (10/14) and 69.2% (9/13) of male and female patients, respectively. There were no significant differences in lymphocyte subsets between male and female patients. With respect to inflammatory factors, levels of CRP (normal range: <1mg/L), serum ferritin (normal range: 30‐400 µg/L for male and 15‐150 µg/L for female), and IL‐2R (normal range: 223‐710 U/mL) were significantly elevated in all patients. The level of IL‐6 (normal range: <7pg/mL) increased in all male patients and in 61.5% of female patients (8/13). TNF‐α (normal range: <8.1 pg/mL) and PCT (normal range: 0.02‐0.05 ng/mL) were both increased in 78.6% (11/14) of male patients and 46.2% (6/13) of female patients. The level of IL‐8 (normal range: <62pg/mL) only increased in 21.4% (3/14) male patients and 15.4% (2/13) of female patients. The level of IL‐10 (normal range: <9.1 pg/mL) was increased in only 42.9% (6/14) of male patients and 30.8% (4/13) of female patients. Pretreatment levels of PCT and TNF‐α were significantly different between male and female patients, but the elevation scale in these patients is not high. Maximum values in male and female patients, respectively, were 0.41 and 0.14 ng/mL for PCT and 17.3 and 11.6 pg/mL for TNF‐α. The level of IL‐1β (normal range: <5 pg/mL) was elevated only in three female patients, with a maximum of 12.2 pg/mL. There was no significant difference in IL‐1β between male and female patients.

**Table 1:** The results of lymphocyte subsets and inflammatory factors between male and female patients (mean ± SD)

	Male (14)	Female (13)	*P*
WBC (×10^9^/L)	5.5 ± 2.1	6.1 ± 2.3	.479
N (×10^9^/L)	4.1 ± 2.1	4.7 ± 2.0	.479
L (×10^9^/L)	0.8 ± 0.4	1.0 ± 0.6	.491
M (×10^9^/L)	0.5 ± 0.2	1.1 ± 2.6	.387
T cell (/µL)	713 ± 442	729 ± 367	.927
B cell (/µL)	147 ± 73	139 ± 92	.819
CD4+ T cell (/µL)	452 ± 267	495 ± 307	.738
CD8+ T cell (/µL)	221 ± 136	216 ± 88	.929
NK cell (/µL)	125 ± 83	108 ± 78	.643
PCT, ng/mL	0.17 ± 0.17	0.06 ± 0.03	**.037**
CRP, mg/L	80.9 ± 66.6	44.2 ± 30.9	.079
IL‐1β, pg/mL	5.0 ± 0.0	6.3 ± 2.5	.093
IL‐2R, U/mL	830.0 ± 288.9	660.6 ± 224.3	.103
IL‐6, pg/mL	44.2 ± 40.3	30.9 ± 37.6	.384
IL‐8, pg/mL	61.5 ± 90.2	28.4 ± 27	.209
IL‐10, pg/mL	11.2 ± 7.5	7.7 ± 3.1	.124
TNF‐α, pg/mL	10.9 ± 3.0	8.0 ± 2.1	**.008**

*Note:* Data are expressed as mean ± SD and were compared using the independent‐samples *t* test.

Abbreviations: CRP, C‐reactive protein; IL‐1β, interleukin‐1β; IL‐2R, IL‐2 receptor; L, lymphocyte; M, monocyte; N, neutrophil; NK, natural killer; PCT, procalcitonin; TNF‐α, tumor necrosis factor‐α; WBC, white blood cell.

*P*  <  .05 was considered as statistically significant.

### 3.2 Most inflammatory factors decreased significantly after treatment

**Figure 1:**
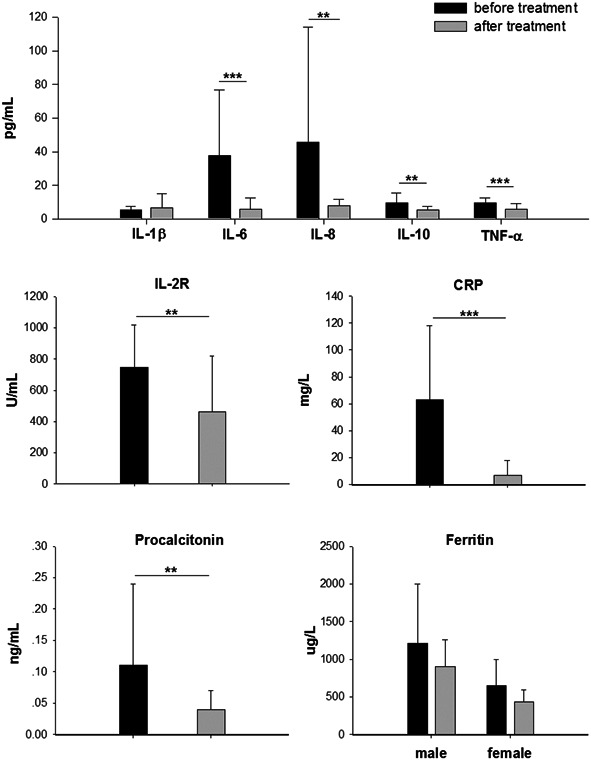
Inflammatory factors in patients with severe COVID‐19 before and after comprehensive treatment. Levels of IL‐1β, IL‐2R, IL‐6, IL‐8, IL‐10, TNF‐α, CRP, PCT, and serum ferritin were measured before and after treatment. Data are expressed as mean ± SD and were compared using the independent‐samples *t* test. COVID‐19, coronavirus disease 2019; CRP, C‐reactive protein; IL‐1β, interleukin‐1β; IL‐2R, IL‐2 receptor; PCT, procalcitonin; TNF‐α, tumor necrosis factor‐α. *P < .05, **P < .01, ***P < .001

Inflammatory factors (IL‐1β, IL‐2R, IL‐6, IL‐8, IL‐10, TNF‐α, CRP, serum ferritin, and PCT) of the 27 patients were compared before and after treatment (Figure 1). After treatment, the respiratory symptoms of all patients were significantly relieved and most of the inflammatory factors were decreased from their pretreatment levels. CRP, IL‐6, TNF‐α, and IL‐2R were significantly decreased after treatment, followed by IL‐8, IL‐10, and PCT. IL‐8 and IL‐10 showed a pretreatment increase in fewer than 50% of the patients. Although PCT was elevated in 63% (17/27) of patients, the maximum level was only 0.41 ng/mL. Levels of IL‐1β and serum ferritin did not change significantly after treatment. In fact, as described above, IL‐1β levels were only elevated slightly in just three female patients. Serum ferritin, however, was elevated in all patients and did not decrease significantly after treatment. It is likely that this inflammatory factor decreased slower than the others.

### 3.3 CD8^+^ and CD4^+^ T lymphocytes were increased significantly after treatment

Lymphopenia is very common in COVID‐19 and is associated with disease severity.^2^, ^11^ In this study, lymphopenia occurs in 70.4% (19/27) of patients. Overall, levels of white blood cells, neutrophils, and monocytes were not significantly different before and after treatment. Pretreatment lymphocyte subsets were analyzed in 21 patients (11 male and 10 female). T lymphocytes (normal range: 955‐2860/µL) were decreased in 76.2% (16/21) of patients, and the minimum value was 210/µL. B lymphocytes (normal range: 90‐560/µL) were decreased by 23.8% (5/21) of patients, with a minimum was 45/µL. CD4^+^ T lymphocytes (normal range: 550‐1440/µL) were decreased by 57.1% (12/21) of patients, and the minimum was 147/µL. CD8^+^ T lymphocytes (normal range: 320‐1250/µL) were decreased in 85.7% (18/21) of patients, and the minimum was 54/µL. NK cells (normal range: 150‐1100/µL) were decreased by 57.1% (12/21) of patients, and the minimum was 27/µL. Lymphopenia improved after treatment in these patients. Among the lymphocyte subsets, the CD8^+^ T lymphocytes showed the most significant improvement, followed by CD4^+^ T lymphocytes. Overall, T lymphocytes and total lymphocytes levels improved significantly. However, the changes in B lymphocytes and NK cells were not significant (Table 2).

**Table 2:** The results of complete blood count and lymphocyte subset before and after treatment (mean ± SD)

	Before treatment	After treatment	*P*
WBC (×109/L)	5.7 ± 2.2	6.6 ± 2.6	.214
N (×10^9^/L)	4.4 ± 2.0	4.4 ± 2.4	.981
L (×10^9^/L)	0.9 ± 0.5	1.5 ± 0.5	**.000**
M (×10^9^/L)	0.7 ± 1.8	0.6 ± 0.2	.696
T cell (/µL)	720 ± 398	1080 ± 341	**.002**
B cell (/µL)	143 ± 80	177 ± 85	.176
CD4+ T cell (/µL)	472 ± 280	678 ± 255	**.012**
CD8+ T cell (/µL)	219 ± 113	355 ± 138	**.001**
NK cell (/µL)	117 ± 79	164 ± 97	.083

*Note:* Data are expressed as mean ± SD and were compared using the independent samples *t* test.

Abbreviations: L, lymphocyte; M, monocyte; N, neutrophil; NK, natural killer; WBC, white blood cell.

*P*  <  .05 was considered as statistically significant.

## 4 DISCUSSION

The COVID‐19 outbreak is a major challenge for clinicians. The disease pathogenesis remains to be fully characterized, and no pharmacologic therapies of proven efficacy yet exist. The immune responses plays important roles in controlling respiratory virus infections.^18^ Distinct patterns of circulating cytokines and acute‐phase responses have proven indispensable in guiding the diagnosis and management of respiratory virus infectious diseases.

Higher levels of proinflammatory cytokines have been associated with lung damage.^19^ IL‐6, IL‐8, and IL‐1β have been reported to contribute to ARDS.^20^ IL‐2R and IL‐6, which appeared to significantly correlate with illness severity by complementing CD8^+^ T cell function,^18^ were presented at significantly higher serum levels in our patients with severe COVID‐19. Although some studies found that the proinflammatory IL‐1 family, including IL‐1β, played an important role in the pathogenesis of COVID‐19,^2^, ^21^, ^22^, ^23^ the level of IL‐1β was normal in most of our patients, and in another study,^24^ the level of IL‐8 was increased in only five patients. This may be related to the severity of the patients recruited in different study. TNF‐α orchestrates the release of chemokines and expression of leukocyte adhesion molecules on the vascular endothelium, promoting the rapid and efficient recruitment of leukocytes toward inflammatory foci.^25^, ^26^ SARS‐CoV infection of dendritic cells induces moderate upregulation of the proinflammatory cytokines TNF and IL‐6.^27^ In our patients with severe COVID‐19, TNF‐α level was increased in most of individuals but by not more than 2.5 fold of the normal range, and decreased significantly after corticosteroid treatment. IL‐10 is the central anti‐inflammatory cytokine.^28^ In our study, IL‐10 levels increased only in 10 patients and decreased significantly after corticosteroid treatment.

CRP plays an important role in innate immunity as an early defense mechanism against infections. Another inflammatory plasma marker that is extensively used in clinical practice is the ferritin. Unlike many bacterial infections, viral infections are commonly characterized by increased circulating ferritin concentrations.^29^, ^30^, ^31^, ^32^, ^33^ In our study, CRP and serum ferritin were increased above normal levels in all patients with severe COVID‐19, but only CRP decreased significantly after treatment. This is probably because serum ferritin levels decreased at a slower rate, but confirmation of this is required. PCT is known for its sensitivity to bacterial infections.^34^ In our investigation, the PCT level was only slightly increased in patients with severe COVID‐19, with the highest level less than 0.5 ng/mL, which does not support bacterial infection. PCT has been investigated for its ability to predict the development of inflammation. However, the clinical effectiveness of this parameter is controversial.^35^, ^36^ In our study, PCT levels decreased significantly after treatment.

In our study, CD4^+^ and particularly CD8^+^ T lymphocyte subtypes were reduced in patients with severe COVID‐19, which is consistent with the general characteristics of viral pneumonia^37^ and reflects the deficiency of the adaptive immune response. Previous research on viral infections has indicated that adaptive T cells, especially CD8^+^ T cells, provide broader and more lasting cross‐reactive cellular immunity with fewer limitations of strain‐specific restriction.^38^ Histological examination of the lungs of patients who have died of COVID‐19 has revealed interstitial mononuclear inflammatory infiltrates, dominated by lymphocytes.^4^ This finding has been correlated with lower CD4^+^ and CD8^+^ T cell counts in the peripheral blood samples of patients with severe COVID‐19. In general, a significantly negative correlation has been reported between the number and function of both CD4^+^ and CD8^+^ T cells.^39^ We found that proinflammatory factors were increased significantly in patients with severe COVID‐19, which may be related to the decrease in T lymphocytes. After treatment, the number of T lymphocytes recovered alongside the decrease in proinflammatory factors. The dynamic changes of lymphocytes function in this process need further study.

It had been reported that COVID‐19 mostly affected men because immune genes are more expressed on the X chromosome. Female patients had lower level of inflammatory factors, while CD4^+^ T cells were higher with better immune response than in male patients with COVID‐19.^40^ But in our patients, except TNF‐α, most of the level of inflammatory factors and the lymphocyte subsets were not significantly different between male and female patients. The differences in the immune response to SARS‐CoV‐2 infection between men and women needs further study.

Although studies have provided evidence that cytokine storms and immunopathology can occur during pathogenic human coronaviruses infections,^5^, ^6^, ^8^, ^9^, ^11^, ^20^, ^41^, ^42^, ^43^, ^44^ we do not yet have a sufficient understanding of the specific factors responsible for exuberant inflammatory responses. Nonetheless, therapeutic interventions targeting these proinflammatory cytokines and chemokines could prove beneficial in ameliorating undesirable inflammatory responses. Corticosteroids are generally used to suppress inflammatory conditions. High‐dose corticosteroids were the mainstay of immunomodulatory therapy during the SARS and MERS epidemics.^45^, ^46^ Corticosteroid administration often leads to early improvements in terms of reduced fever, resolution of radiographic lung infiltrates, and better oxygenation.^47^, ^48^, ^49^ However, some studies have shown no beneficial effect of corticosteroids, while others have demonstrated adverse outcomes following corticosteroid therapy. Some experts believed there is no clinical data to indicate that a net benefit is derived from corticosteroids in treating respiratory infection due to SARS‐CoV or MERS‐CoV, and so corticosteroid treatment should not be used for the treatment of COVID‐19‐induced lung injury or shock outside of a clinical trial.^50^ Despite this, corticosteroids were used in patients with severe COVID‐19 in China,^2^, ^4^ but the timing, dose, and duration of corticosteroid therapy may be critical. In the patients included in our study, corticosteroids were only used when the patient met the criteria for severe pneumonia, including those whose pulmonary imaging progressed greater than 50% in 24 to 48 hours. In the current diagnosis and treatment protocol, the diagnosis of mild and severe patients with COVID‐19 is mainly based on clinical symptoms, signs and pulmonary imaging. It had been reported that the difference of the level of inflammation factors, particularly IL‐2R, IL‐6 and TNF‐α, between patients with mild and severe COVID‐19 was significant.^24^ In our study, the level of the cytokines above was also decreased significantly after the treatment with corticosteroid. These findings suggested that the level of inflammatory factors may be a more objective indicator to distinguish mild and severe patients with COVID‐19 and to determine the timing, dosage, and course of corticosteroid therapy. However, the cutoff value of these inflammatory factors needs further study.

Some limitations of this study should also be acknowledged. This was a retrospective, single‐center, observational study, and unavoidable biases occurred when including participants. Furthermore, the sample size was very small. Despite these limitations, the study reflects the “real life” clinical situation.

In conclusion, a proinflammatory response, particularly the level of IL‐2R, IL‐6, TNF‐α, and CRP, were associated with severe COVID‐19. The SARS‐CoV‐2 infection affect primarily T lymphocyte, particularly CD8^+^ T cells. The lymphocytes function in patients with severe COVID‐19 need to be further clarified.

## CONFLICT OF INTERESTS

The authors declare that there are no conflict of interests.

## AUTHOR CONTRIBUTIONS

MN researched literature and wrote the first draft of the manuscript. F‐BT and D‐DX collected the patients’ clinical data. BY designed the investigation, and reviewed and modified the manuscript. All authors have read and approved the final version of the manuscript.

## ETHICS STATEMENT

This study received ethical approval from the Medical Ethics Committee of Tongji Hospital, Tongji Medical College, Huazhong University of Science and Technology. Written informed consent for publication of these clinical details was obtained from each patient. Copies of the consent form are available for review by the editor of this journal.

## Data Availability

The data used during the current report are available from the corresponding author on reasonable request.

## References

[1] Heymann DL , Shindo N . COVID‐19: what is next for public health? Lancet. 2020;395:542‐545. 32061313 10.1016/S0140-6736(20)30374-3 PMC7138015

[2] Huang C , Wang Y , Li X , et al. Clinical features of patients infected with 2019 novel coronavirus in Wuhan, China. Lancet. 2020;395:497‐506. 31986264 10.1016/S0140-6736(20)30183-5 PMC7159299

[3] Wu Z , McGoogan JM . Characteristics of and important lessons from the coronavirus disease 2019 (COVID‐19) outbreak in China: summary of a report of 72314 cases from the Chinese Center for Disease Control and Prevention. JAMA. 2020;323:1239‐1242. 10.1001/jama.2020.2648 32091533

[4] Xu Z , Shi L , Wang Y , et al. Pathological findings of COVID‐19 associated with acute respiratory distress syndrome. Lancet Respir Med. 2020;8:420‐422. 32085846 10.1016/S2213-2600(20)30076-X PMC7164771

[5] Ding Y , Wang H , Shen H , et al. The clinical pathology of severe acute respiratory syndrome (SARS): a report from China. J Pathol. 2003;200:282‐289. 12845623 10.1002/path.1440 PMC7168017

[6] Ng DL , Al Hosani F , Keating MK , et al. Clinicopathologic, immunohistochemical, and ultrastructural findings of a fatal case of Middle East respiratory syndrome coronavirus infection in the United Arab Emirates, April 2014. Am J Pathol. 2016;186:652‐658. 26857507 10.1016/j.ajpath.2015.10.024 PMC7093852

[7] Cameron MJ , Bermejo‐Martin JF , Danesh A , Muller MP , Kelvin DJ . Human immunopathogenesis of severe acute respiratory syndrome (SARS). Virus Res. 2008;133:13‐19. 17374415 10.1016/j.virusres.2007.02.014 PMC7114310

[8] Channappanavar R , Perlman S . Pathogenic human coronavirus infections: causes and consequences of cytokine storm and immunopathology. Semin Immunopathol. 2017;39:529‐539. 28466096 10.1007/s00281-017-0629-x PMC7079893

[9] Min CK , Cheon S , Ha NY , et al. Comparative and kinetic analysis of viral shedding and immunological responses in MERS patients representing a broad spectrum of disease severity. Sci Rep. 2016;6:25359. 27146253 10.1038/srep25359 PMC4857172

[10] Williams AE , Chambers RC . The mercurial nature of neutrophils: still an enigma in ARDS? Am J Physiol Lung Cell Mol Physiol. 2014;306:L217‐L230. 24318116 10.1152/ajplung.00311.2013 PMC3920201

[11] Chen N , Zhou M , Dong X , et al. Epidemiological and clinical characteristics of 99 cases of 2019 novel coronavirus pneumonia in Wuhan, China: a descriptive study. Lancet. 2020;395:507‐513. 32007143 10.1016/S0140-6736(20)30211-7 PMC7135076

[12] Hotchkiss RS , McConnell KW , Bullok K , et al. TAT‐BH4 and TAT‐Bcl‐xL peptides protect against sepsis‐induced lymphocyte apoptosis in vivo. J Immunol. 2006;176:5471‐5477. 16622015 10.4049/jimmunol.176.9.5471

[13] Hotchkiss RS , Osmon SB , Chang KC , Wagner TH , Coopersmith CM , Karl IE . Accelerated lymphocyte death in sepsis occurs by both the death receptor and mitochondrial pathways. J Immunol. 2005;174:5110‐5118. 15814742 10.4049/jimmunol.174.8.5110

[14] Hotchkiss RS , Tinsley KW , Swanson PE , et al. Sepsis‐induced apoptosis causes progressive profound depletion of B and CD4+ T lymphocytes in humans. J Immunol. 2001;166:6952‐6963. 11359857 10.4049/jimmunol.166.11.6952

[15] Jensen IJ , Winborn CS , Fosdick MG , et al. Polymicrobial sepsis influences NK‐cell‐mediated immunity by diminishing NK‐cell‐intrinsic receptor‐mediated effector responses to viral ligands or infections. PLoS Pathog. 2018;14:e1007405. 30379932 10.1371/journal.ppat.1007405 PMC6231673

[16] Guan W , Ni Z , Hu Y , et al. Clinical characteristics of coronavirus disease 2019 in China. N Engl J Med. 2020;382:1708‐1720. 32109013 10.1056/NEJMoa2002032 PMC7092819

[17] National Health Commission of China. 2020. Diagnosis and treatment program of new coronavirus pneumonia (trial version 7).

[18] Nüssing S , Sant S , Koutsakos M , Subbarao K , Nguyen THO , Kedzierska K . Innate and adaptive T cells in influenza disease. Front Med. 2018;12(1):34‐47. 10.1007/s11684-017-0606-8 29352371

[19] Das D , Le Floch H , Houhou N , et al. Viruses detected by systematic multiplex polymerase chain reaction in adults with suspected community‐acquired pneumonia attending emergency departments in France. Clin Microbiol Infect. 2015;21:608.e1‐e8. 25704448 10.1016/j.cmi.2015.02.014 PMC7128919

[20] Jiang Y , Xu J , Zhou C , et al. Characterization of cytokine/chemokine profiles of severe acute respiratory syndrome. Am J Respir Crit Care Med. 2005;171:850‐857. 15657466 10.1164/rccm.200407-857OC

[21] Conti P , Gallenga CE , Tetè G , et al. How to reduce the likelihood of coronavirus‐19 (CoV‐19 or SARS‐CoV‐2) infection and lung inflammation mediated by IL‐1 [published online ahead of print March 31, 2020]. J Biol Regul Homeost Agents. 2020;34(2). 10.23812/Editorial-Conti-2 32228825

[22] Conti P , Ronconi G , Caraffa A , et al. Induction of pro‐inflammatory cytokines (IL‐1 and IL‐6) and lung inflammation by coronavirus‐19 (COVI‐19 or SARS‐CoV‐2): anti‐inflammatory strategies. J Biol Regul Homeost Agents. 2020;34(2). 10.23812/CONTI-E 32171193

[23] Kritas SK , Ronconi G , Caraffa A , Gallenga CE , Ross R , Conti P . Mast cells contribute to coronavirus‐induced inflammation: new anti‐inflammatory strategy [published online ahead of print February 4, 2020]. J Biol Regul Homeost Agents. 2020;34(1). 10.23812/20-Editorial-Kritas 32013309

[24] Chen G , Wu D , Guo W , et al. Clinical and immunological features of severe and moderate coronavirus disease 2019. J Clin Invest. 2020;130:2620‐2629. 32217835 10.1172/JCI137244 PMC7190990

[25] JR Bradley . TNF‐mediated inflammatory disease. J Pathol. 2008;214(2):149‐160. 10.1002/path.2287 18161752

[26] Wang X , Feuerstein GZ , Gu JL , Lysko PG , Yue TL . Interleukin‐1 beta induces expression of adhesion molecules in human vascular smooth muscle cells and enhances adhesion of leukocytes to smooth muscle cells. Atherosclerosis. 1995;115(1):89‐98. 10.1016/0021-9150(94)05503-b 7545398

[27] Cheung CY , Poon LLM , Ng IHY , et al. Cytokine responses in severe acute respiratory syndrome coronavirus‐infected macrophages in vitro: possible relevance to pathogenesis. J Virol. 2005;79:7819‐7826. 15919935 10.1128/JVI.79.12.7819-7826.2005 PMC1143636

[28] Couper KN , Blount DG , Riley EM . IL‐10: the master regulator of immunity to infection. J Immunol. 2008;180:5771‐5777. 18424693 10.4049/jimmunol.180.9.5771

[29] Slaats J , Ten Oever J , van de Veerdonk FL , Netea MG . IL‐1beta/IL‐6/CRP and IL‐18/ferritin: distinct inflammatory programs in infections. PLoS Pathog. 2016;12:e1005973. 27977798 10.1371/journal.ppat.1005973 PMC5158075

[30] Soundravally R , Agieshkumar B , Daisy M , Sherin J , Cleetus CC . Ferritin levels predict severe dengue. Infection. 2015;43(1):13‐19. 10.1007/s15010-014-0683-4 25354733

[31] van de Weg CAM , Huits RMHG , Pannuti CS , et al. Hyperferritinaemia in dengue virus infected patients is associated with immune activation and coagulation disturbances. PLoS Negl Trop Dis. 2014;8:e3214. 25299654 10.1371/journal.pntd.0003214 PMC4191960

[32] Wang W , Knovich MA , Coffman LG , Torti FM , Torti SV . Serum ferritin: past, present and future. Biochim Biophys Acta. 2010;1800:760‐769. 20304033 10.1016/j.bbagen.2010.03.011 PMC2893236

[33] Wu J , Chen L , Chen Y , Yang J , Wu D . Serum ferritin concentration predicts mortality in patients with hepatitis B virus‐related acute on chronic liver failure. Arch Med Res. 2014;45(3):251‐256. 10.1016/j.arcmed.2014.03.004 24656903

[34] Covington EW , Roberts MZ , Dong J . Procalcitonin monitoring as a guide for antimicrobial therapy: a review of current literature. Pharmacotherapy. 2018;38(5):569‐581. 10.1002/phar.2112 29604109

[35] Kim BG , Noh MH , Ryu CH , et al. A comparison of the BISAP score and serum procalcitonin for predicting the severity of acute pancreatitis. Korean J Intern Med. 2013;28:322‐329. 23682226 10.3904/kjim.2013.28.3.322 PMC3654130

[36] Simsek O , Kocael A , Kocael P , et al. Inflammatory mediators in the diagnosis and treatment of acute pancreatitis: pentraxin‐3, procalcitonin and myeloperoxidase. Arch Med Sci. 2018;14:288‐296. 29593801 10.5114/aoms.2016.57886 PMC5868652

[37] Guo L , Wei D , Zhang X , et al. Clinical features predicting mortality risk in patients with viral pneumonia: the MuLBSTA score. Front Microbiol. 2019;10:2752. 31849894 10.3389/fmicb.2019.02752 PMC6901688

[38] Bender BS , Croghan, T , Zhang L , Small PA Jr. Transgenic mice lacking class I major histocompatibility complex‐restricted T cells have delayed viral clearance and increased mortality after influenza virus challenge. J Exp Med. 1992;175(4):1143‐1145. 10.1084/jem.175.4.1143 1552285 PMC2119177

[39] Luo Y , Xie Y , Zhang W , et al. Combination of lymphocyte number and function in evaluating host immunity. Aging. 2019;11(24):12685‐12707. 10.18632/aging.102595 31857499 PMC6949078

[40] Conti P , Younes A . Coronavirus COV‐19/SARS‐CoV‐2 affects women less than men: clinical response to viral infection [published online ahead of print April 7, 2020]. J Biol Regul Homeost Agents. 2020;34(2). 10.23812/Editorial-Conti-3 32253888

[41] Chen J , Subbarao K . The immunobiology of SARS*. Annu Rev Immunol. 2007;25:443‐472. 17243893 10.1146/annurev.immunol.25.022106.141706

[42] Chousterman BG , Swirski FK , Weber GF . Cytokine storm and sepsis disease pathogenesis. Semin Immunopathol. 2017;39:517‐528. 28555385 10.1007/s00281-017-0639-8

[43] Fung TS , Liu DX . Human coronavirus: host‐pathogen interaction. Annu Rev Microbiol. 2019;73:529‐557. 31226023 10.1146/annurev-micro-020518-115759

[44] Mahallawi WH , Khabour OF , Zhang Q , Makhdoum HM , Suliman BA . MERS‐CoV infection in humans is associated with a pro‐inflammatory Th1 and Th17 cytokine profile. Cytokine. 2018;104:8‐13. 29414327 10.1016/j.cyto.2018.01.025 PMC7129230

[45] Stockman LJ , Bellamy R , Garner P . SARS: systematic review of treatment effects. PLoS Med. 2006;3(9):e343. 10.1371/journal.pmed.0030343 16968120 PMC1564166

[46] Arabi YM , Mandourah Y , Al‐Hameed F , et al. Corticosteroid therapy for critically Ill patients with Middle east respiratory syndrome. Am J Respir Crit Care Med. 2018;197(6):757‐767. 10.1164/rccm.201706-1172OC 29161116

[47] Auyeung TW , Lee JSW , Lai WK , et al. The use of corticosteroid as treatment in SARS was associated with adverse outcomes: a retrospective cohort study. J Infect. 2005;51(2):98‐102. 10.1016/j.jinf.2004.09.008 16038758 PMC7132384

[48] Ho JC , OOi GC , Mok TY , et al. High‐dose pulse versus nonpulse corticosteroid regimens in severe acute respiratory syndrome. Am J Respir Crit Care Med. 2003;168(12):1449‐1456. 10.1164/rccm.200306-766OC 12947028

[49] Yam LY‐C , Lau AC‐W , Lai Y‐LF , et al. Corticosteroid treatment of severe acute respiratory syndrome in Hong Kong. J Infect. 2007;54(1):28‐39. 10.1016/j.jinf.2006.01.005 16542729 PMC7112522

[50] Russell CD , Millar JE , Baillie JK . Clinical evidence does not support corticosteroid treatment for 2019‐nCoV lung injury. Lancet. 2020;395:473‐475. 32043983 10.1016/S0140-6736(20)30317-2 PMC7134694

